# Spectral analysis of gastric aspirates obtained shortly after birth predicts the need for prolonged respiratory support in neonates in a development cohort

**DOI:** 10.3389/fped.2025.1686794

**Published:** 2025-12-11

**Authors:** Brianna C. Leigh, Lavonne M. Liedl, Amy L. Amsbaugh, William A. Carey

**Affiliations:** 1 Division of Neonatal Medicine, Mayo Clinic, Rochester, MN, United States; 2 Department of Anesthesiology and Perioperative Medicine, Mayo Clinic, Rochester, MN, United States

**Keywords:** newborn infant, neonatal respiratory distress syndrome, transient tachypnea of the newborn, point-of-care diagnostics, artificial intelligence, machine learning, respiratory therapy

## Abstract

**Introduction:**

Spectral analysis of gastric aspirates obtained shortly after birth predicts the diagnosis of respiratory distress syndrome in neonates born <32 completed weeks gestation. We sought to determine whether this prototype point-of-care device measuring surfactant components in gastric aspirates could predict prolonged respiratory support needs in neonates ≥30 completed weeks gestation.

**Methods:**

Gastric aspirates obtained within 30 min of birth were analyzed by spectroscopy to quantify surfactant components. These spectral data were entered into an existing algorithm to assess subjects' biochemical lung maturity. This algorithmic output was paired with clinical data to evaluate the performance of the algorithm in predicting subjects' need for respiratory support at six hours of life (prolonged respiratory support). Each element of the algorithm was adjusted via a machine learning framework to optimize predictive performance.

**Results:**

Gastric aspirates from 179 subjects (median 36 weeks, range 31–41 weeks) were eligible for analysis. Spectral analysis of gastric aspirates predicted the need for prolonged respiratory support with 70% sensitivity and 92% specificity. Positive- and negative-predictive values were 86% and 82%, respectively, for the overall cohort. Among gestational age subgroups, positive prediction was highest among moderately preterm neonates (32–33 weeks), while negative prediction was highest among term neonates.

**Discussion:**

Spectral analysis of surfactant components contained in the gastric fluid of neonates ≥30 completed weeks gestation predicts the need for prolonged respiratory support with good performance. Predictive performance varied according to subjects' gestational age at birth, suggesting that gestational age-specific algorithms may improve the performance of this point-of-care diagnostic test.

## 1 Introduction

Respiratory distress at the time of birth may be due to a variety of etiologies, with premature neonates affected more often than term neonates (1–3). While national guidelines permit lower-level nurseries to admit neonates as immature as 32 weeks gestation, they also restrict pressure-supported respiratory therapies, such as continuous positive airway pressure (CPAP), to neonates whose respiratory distress is “expected to resolve rapidly” (4). Because of the diagnostic uncertainties of neonatal respiratory distress (5–9), it is challenging for pediatricians in community settings to determine whether to admit an affected neonate to their own lower-level nursery or transfer the patient to a neonatal intensive care unit (NICU).

Surfactant deficiency is a leading cause of neonatal respiratory distress. It is well characterized as the primary cause of respiratory distress syndrome (RDS), and it also may contribute to the pathophysiology of transient tachypnea of the newborn (TTN) and other transitional physiologic states (10). As the fetal lung matures over the course of gestation, the amount of surfactant present in fetal lung fluid increases. Because fetal lung fluid is swallowed *in utero*, surfactant is present in the gastric fluid of mature neonates at the time of delivery and may be quantified in gastric aspirates (GAs) obtained shortly after delivery (10–13).

Recent evidence suggests that spectroscopic quantification of GA surfactant components predicts the diagnosis of RDS in neonates <32 completed weeks gestation (14). When samples of gastric fluid were obtained within 45 min of birth, Fourier Transform Infrared spectroscopy (FTIR)-quantification of lecithin (L) and sphingomyelin (S) permitted investigators to use the L/S ratio to predict RDS with a high degree of certainty (14). While these results are promising, it remains to be seen whether similar diagnostic precision would be achieved in more mature neonates. Thus, in the context of the American system of neonatal care and the guidelines informing that care (4), we designed the current study to determine whether FTIR-analysis of GAs could predict the need for prolonged respiratory support in neonates >30 completed weeks gestation.

## 2 Methods

### 2.1 Study approval

This research protocol was approved by the Mayo Clinic institutional review board.

### 2.2 Eligibility criteria

Neonates born ≥30 completed weeks gestation who required orogastric suctioning within 30 min of birth were eligible for this study. We excluded neonates with known or suspected congenital anomalies, those for whom only comfort measures were planned, and those who received exogenous surfactant therapy prior to obtaining the gastric aspirate. Neonates with other identifiable causes of respiratory distress including pneumothorax, meconium aspiration syndrome, or pneumonia were excluded.

### 2.3 Setting

This study was conducted in the labor and delivery unit in our hospital. While our institution operates a high-risk, regional perinatal center, it also provides routine obstetric care for expectant mothers who live in the community. In fact, most neonates delivered at Mayo Clinic are admitted to the well-baby (Level I) nursery. Neonates who do require respiratory support following resuscitation, and those who develop respiratory distress after admission to the well-baby nursery, are admitted to one of our two NICUs for evaluation and management. Because the NICU nearest our labor and delivery unit routinely admits patients ≥30 weeks gestation, we planned to enroll subjects down to this level of prematurity.

### 2.4 Personnel

Gastric aspirate samples were collected by members of the neonatal resuscitation team as part of their routine delivery room care. The authors of this manuscript formed the research team and were the only personnel with access to subjects' demographic and clinical data. The manufacturer of the FTIR device, SIME Dx (London, UK), provided the device, consumable materials, and engineers capable of processing and analyzing GAs. Both the research team and SIME engineers had access to the FTIR-derived data.

### 2.5 Gastric aspirate collection

Samples of gastric fluid were collected during the course of routine delivery room resuscitation at our hospital. In this setting, orogastric suctioning is clinically indicated to relieve excess air that accumulates during resuscitation and/or swallowed secretions that may reflux into the oropharynx. Gastric aspirates thus were collected without altering the care provided by the neonatal resuscitation team.

After completing all care associated with the delivery room resuscitation, a member of the clinical team would enter the subjects' names and medical record numbers into a logbook that contained sequentially numbered pages. These page numbers served as unique subject-identification numbers for subsequent analyses, thereby protecting subjects' anonymity outside the research team.

### 2.6 Clinical care and respiratory support assessment

As was the case for the delivery room resuscitations, all clinical decisions were made by the clinicians on the neonatal team without knowledge of the FTIR-derived data. The clinical team determined which nursery was most appropriate for each subject and made all patient-care decisions, including those related to respiratory support.

Because lower-level nurseries may care for respiratory conditions that are “expected to resolve rapidly,” we assessed subjects' respiratory care requirements at the time of admission and at six hours of life. We selected this time point based on our experience with the medical transport of neonates in our region: most transport requests come within an hour or two of birth, and most others occur after a two- to six-hour period of management in a lower-level nursery. Respiratory care requirements were recorded as room air; supplemental oxygen (either isolette or low-flow nasal cannula oxygen); nasal CPAP or intermittent positive-pressure ventilation (NIPPV); or mechanical ventilation (conventional or high-frequency).

### 2.7 Analysis and machine learning of surfactant components

Gastric aspirate samples were stored at room temperature for twice-daily collection by the engineer who would process the GA for analysis of surfactant components. As previously reported by Schousboe et al. (15), 100 mcl aliquots of GA were diluted four-fold with water and centrifuged at 4,000 *g* for four minutes. Following removal of the supernatant, the pellet was resuspended in 100 mcl water. This sample was loaded into an automated device that deposited the sample on a CaF2 window, where it was heated and dried to isolate lamellar bodies in order to obtain FTIR spectra of the desiccated biochemicals.

### 2.8 Development of an algorithm to predict prolonged respiratory support

**Figure 1:**
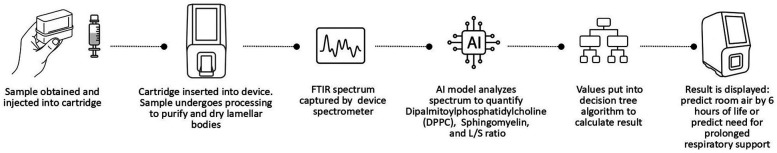
Illustration of workflow from gastric aspirate sample collection to result from POC device.

Because most out born neonates are transferred to our hospital within six hours of delivery, we defined *prolonged respiratory support* as the need for any form of respiratory support at six hours of life. This dichotomous outcome then was compared to the outcome predicted by an existing algorithm that was optimized for neonates <32 completed weeks gestation (14). As previously described by Schousboe et al. [Appendix (14),], we then adjusted the FTIR-quantified lecithin as dipalmitoyl phosphatidylcholine (DPPC), sphingomyelin, and the resulting L/S ratio outputs to optimize predictive performance among all subjects. The quantitative machine learning model used was derived using Projection to Latent Structures (PLS) models (16, 17). To reduce the risk of overfitting during training of the model, cross-validation was applied in predicting lecithin and sphingomyelin levels. The results of these outputs were put into a decision tree algorithm (18) which then gave a result of predicting need for prolonged respiratory support >6 h or predicting room air by 6 h of life. We further assessed the performance of this optimized algorithm in each gestational age subgroup represented in our overall cohort (very preterm, 30–31 completed weeks gestation; moderately preterm, 32–33 weeks; late-preterm, 34–36 weeks; and term, ≥37 weeks) (19). Figure 1 displays the workflow from sample collection to result from the device.

### 2.9 Statistical analysis

A traditional power calculation was not performed, as the objective of this study was to evaluate if the developed AI algorithm achieved acceptable predictive performance rather than to test a predefined hypothesis or compare intervention groups. Accordingly, the sample size was selected to provide sufficient precision in estimating performance metrics and ensure adequate representation of the target clinical population.

Using paired spectral and clinical data, we determined the performance of the algorithm in predicting patients' need for prolonged respiratory support by performing descriptive statistics and calculating sensitivity, specificity, positive predictive value (PPV), and negative predictive value (NPV). Ninety-five percent confidence intervals were calculated for each, using the Clopper-Pearson Method for sensitivity and specificity, and Wilson Score Interval Method for PPV and NPV. The ROC curve and optimal decision threshold were validated using the method described by DeLong et al. (20).

## 3 Results

### 3.1 Enrollment and sample accrual

**Figure 2:**
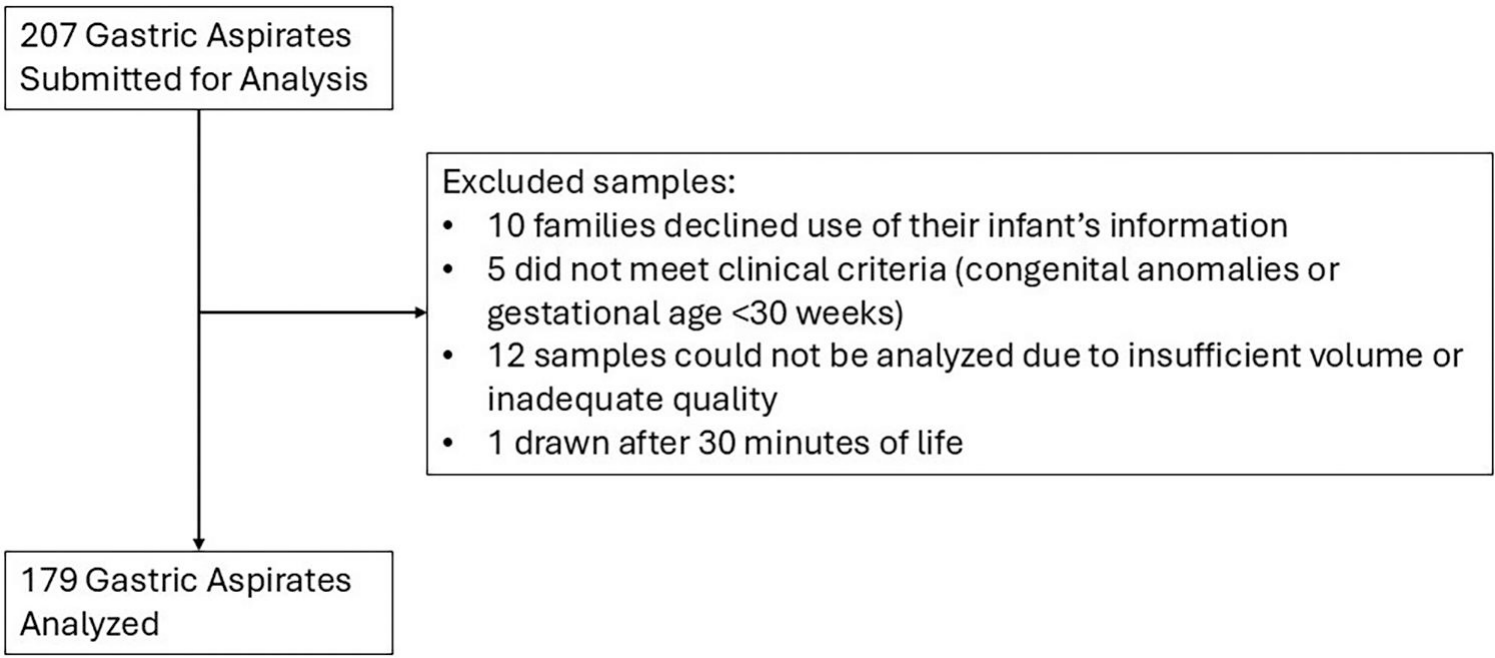
Flow diagram demonstrating reasons for excluding samples collected for analysis.

Over a nine-month period (June 2024–February 2025) we collected GAs from 207 neonates (median completed weeks gestation 36 weeks, range 31–41 weeks). As shown in Figure 2, 28 samples were excluded from analysis for reasons related to data use authorization, adherence to protocol, and sample characteristics. The remaining 179 samples were analyzed as described above, and the demographic characteristics of those subjects are shown in Table 1.

**Table 1:** Maternal and neonatal demographic characteristics.

Characteristic	% (*n* = 179)
Maternal race	
Black	8% (14)
White	69% (123)
Other	7% (12)
Unknown	16% (30)
Maternal diabetes	28% (44)
Maternal smoke/tobacco use	7% (10)
Antenatal steroids	45% (66)
Cesarean delivery	76% (121)
Male sex	56% (101)
Small for gestational age	12% (22)
Median gestational age at birth, weeks (IQR)	36 (33–38)
Median birthweight, grams (IQR)	2,640 (2,140–3,230)

Among the 179 subjects whose GAs were analyzed, 71 required at least some form of respiratory support at six hours of life. As might be expected due to the gestational age range of our subjects, the vast majority (67) were supported with CPAP at six hours, with mechanical ventilation (3) and NIPPV (1) being less common. Given that we analyzed surfactant components in our subjects' GAs, we note here that 11 subjects were intubated for treatment with surfactant therapy by 24 h of life. No infants were diagnosed with pneumothorax, meconium aspiration syndrome, or pneumonia.

### 3.2 Optimization of predictive algorithm

Adjustment of the lecithin, sphingomyelin, and L/S ratios for each subject yielded a new algorithm that predicted the need for prolonged respiratory support with reasonably good performance. As shown in Table 2, analysis of GAs obtained within 30 min of delivery predicted the need for respiratory support at six hours of life with 70% sensitivity and 92% specificity. In our study population, these characteristics yielded positive- (PPV) and negative-predictive values (NPV) 86% and 82% respectively. The area under the curve for the entire cohort was 81% (95% CI: 74%–88%).

**Table 2:** Predictive performance of algorithm.

Subjects	Sensitivity (95% CI: lower limit, upper limit)	Specificity (95% CI: lower limit, upper limit)	Positive Predictive Value (95% CI: lower limit, upper limit)	Negative Predictive Value (95% CI: lower limit, upper limit)
Infants 31–41 completed weeks gestation (*n* = 179)	70% (95% CI: 58%, 80%)	92% (95% CI: 86%, 97%)	86% (95% CI: 75%, 93%)	82% (95% CI: 77%, 90%)
Gestational age subgroups				
31 (*n* = 6)	83% (95% CI: 36%, 100%)	Unable to calculate	100% (95% CI: 57%, 100%)	Unable to calculate
32–33 (*n* = 42)	79% (95% CI: 61%, 91%)	89% (95% CI: 52%, 100%)	96% (95% CI: 82%, 99%)	53% (95% CI: 43%, 86%)
34–36 (*n* = 54)	62% (95% CI: 41%, 80%)	86% (95% CI: 67%, 96%)	80% (95% CI: 58%, 92%)	71% (95% CI: 61%, 88%)
≥37 (*n* = 76)	50% (95% CI: 16%, 84%)	96% (95% CI: 88%, 99%)	57% (95% CI: 25%, 84%)	94% (95% CI: 87%, 98%)

CI, confidence interval.

### 3.3 Gestational age-specific predictive performance

As described above, we optimized an algorithm to predict the need for prolonged respiratory support in a developmentally broad spectrum of patients (31–41 completed weeks gestation). We next sought to determine the predictive performance of this algorithm within established gestational age subgroups (19). As shown in Table 2, sensitivity was inversely related to gestational age, while specificity was relatively high in each subgroup.

### 3.4 Prediction of surfactant therapy

As might be expected due to our study design, only 11 subjects ultimately received treatment with surfactant. The optimized algorithm predicted surfactant deficiency in 9 of the 11 subjects, whose gestational ages ranged 32–37 completed weeks gestation.

## 4 Discussion

Community-level hospitals face specific challenges in the management of neonatal respiratory distress at the time of delivery. While lower-level nurseries may admit neonates at high risk for RDS (e.g., preterm neonates, infants of diabetic mothers) or those with clinically apparent respiratory distress of any cause, they are limited in the modes and duration of respiratory support they may provide to their patients (4). This circumstance is made even more complex by the ambiguity of clinical and radiographic assessment of respiratory distress in most neonates born ≥32 weeks gestation (5–9). To address this problem, we measured surfactant components in samples of gastric fluid obtained within the first 30 min of life, then analyzed these data in a machine learning framework to predict the need for respiratory support at six hours of life. The analysis of surfactant components in gastric fluid demonstrates strong predictive ability in determining the need for prolonged respiratory support. The predictive accuracy varied with gestational age at birth, suggesting gestational-age specific algorithms could further enhance the performance.

### 4.1 Subjects—demographic and clinical characteristics

Our subjects' demographic and clinical characteristics were as expected for a regional perinatal center in the state of Minnesota (21). Among the 149 subjects whose maternal race was known, 83% were White, 9% were Black, and 8% were from other backgrounds. Given the high-risk population of our obstetrics practice, our subjects' mothers were more likely to report smoking during pregnancy, require treatment for diabetes, and deliver via Caesarian section than the overall population in Minnesota and the United States (21, 22). Of the infants who were included in the analysis, the median gestational age at birth was 36 weeks (range 31–41 weeks) and median birth weight was 2,640 g (range 1,280–4,690 g).

### 4.2 Positive predictive performance—respiratory support ≥6 h

Community-level hospitals are increasingly initiating nasal CPAP to treat neonatal respiratory distress (23), perhaps in part due to the rise in the rate of Caesarian delivery and the adverse respiratory outcomes associated with it (24, 25). Given the uncertainty in discriminating some etiologies of neonatal respiratory distress (5–9), pediatricians are unable to determine whether a given patient's CPAP requirement will “resolve rapidly” as required by levels-of-care guidelines (1). For neonates with RDS, this diagnostic ambiguity leads to delays in the initiation of CPAP support when indicated which increases the risk of adverse complications, such as air leak (26, 27).

In the present study, we adapted the approach of Schousboe et al. (14, 15) to determine whether FTIR spectrometry would permit early identification of neonates who would require respiratory support for more than six hours—that is, whether it could “rule in” prolonged respiratory support. Among the overall cohort, which was comprised mostly of term (42%) and late-preterm (30%) neonates, optimization of the algorithm yielded a PPV of 86%. By shifting the paradigm from subjective clinical assessment to objective biochemical analysis, this approach may improve the timeliness and outcomes of respiratory therapy in this population.

### 4.3 Negative predictive performance—respiratory support <6 h

Not all neonates with respiratory distress at the time of birth will go on to require prolonged respiratory support. Given that clinical and radiographic assessments of neonatal respiratory distress are notoriously unreliable (5–9), some community-based pediatricians may err on the side of caution and transfer a distressed neonate to the NICU within one or two hours. This scenario leads to “over-treatment” of neonatal respiratory distress in some cases, with the potential for separation of the maternal-neonatal dyad, inherent safety risks of medical transport, and the incurrence of additional health care cost (28, 29).

Given the need for an objective means to “rule out” a requirement for prolonged respiratory support, we calculated the negative-predictive value of the cohort-optimized algorithm. Spectral analysis of GAs obtained within 30 min of delivery correctly identified whether a patient would breathe room air at six hours of life in 82% of cases. This objective information could enable community-based pediatricians to make management decisions, including whether to transfer/transport to the NICU, with greater accuracy and confidence.

### 4.4 Gestational age-specific predictive performance

While we optimized the algorithm for use in the overall cohort (31–41 completed weeks gestation), we then assessed its predictive performance in each of the gestational age subgroups. Among the moderately preterm, late-preterm, and term neonates, PPV and NPV were inversely and directly correlated with gestational age (Table 2). This may relate to the fact that PPV and NPV are influenced by the prevalence of disease in a given population. Because premature neonates are more likely to require prolonged respiratory support (1–3), it was not surprising to find that PPV was highest among moderately preterm neonates and that NPV was highest among term neonates. The lower NPV observed in our less mature subjects also could reflect respiratory distress due to factors other than surfactant deficiency (30), such as increased chest wall compliance and/or relative weakness of diaphragmatic excursion. Future studies of this technology could elucidate whether gestational age-specific algorithms could yield better positive- and negative-predictive performance when optimized according to gestational age.

### 4.5 Alternatives to FTIR-based diagnosis

Other investigators have studied more objective means of assessing neonatal respiratory distress, with most relating to the specific diagnosis of RDS. Biochemical tests to predict RDS unfortunately have performed with mixed results (31, 32). Lung ultrasound has been studied to diagnose RDS and predict which infants would benefit from exogenous surfactant treatment (33–37). While it has been more widely adopted by neonatologists in Europe, its use in the United States remains limited. The American Academy of Pediatrics acknowledges that lung ultrasound can discriminate between TTN and RDS, however it notes that its application in the US thus far has been hindered by insufficient training, limited collaboration with imaging specialists, and concerns about litigation. Consequently, although lung ultrasound could potentially address the same challenges as the machine learning-based approach described in this study, it has yet to achieve its full potential within American neonatology (38). Lung ultrasound also may not be feasible or easily accessible in hospitals with relatively low-level nursery capabilities. On the other hand, most newborn infants requiring respiratory support will have gastric decompression performed. Given the low volume of gastric fluid required for FTIR analysis of surfactant components (100 mcL), we suspect that this technique would be feasible and desirable for clinicians and families if its precision is proved in future studies.

### 4.6 Limitations

The initial algorithm used in this study was derived from preterm neonates born in Denmark. The demographics of that population are quite different than those of the United States, as a higher proportion of Denmark's population is of Danish descent (39). There are known differences in respiratory outcomes of preterm infants based on race which may or may not be affected by surfactant concentrations (40–43). There also may be difference reference ranges depending on demographic features such as race, ethnicity, and gender (44, 45) which are not currently accounted for in the algorithm.

Not all patients from whom we obtained GAs were included in this study. Ten mothers (5%) declined to authorize our use of their and/or their children's clinical data. It is conceivable that these mothers were more likely to have characteristics associated with higher risk of neonatal respiratory distress, and/or perceived their children to be “too sick,” than those who did not decline. This scenario could skew the cohort toward a lower prevalence of prolonged respiratory support, which would reduce the positive-predictive performance, especially in more mature neonates. We also could not analyze some samples (6%) due to inadequate GA volume or quality. This may reflect the effects of evaporation or other physical process that occurred between the time of sample acquisition and our attempted analysis. This problem may be avoided in future studies with location of the FTIR device at the bedside as point-of-care testing.

### 4.7 Future directions

Having developed a predictive algorithm among a cohort comprised mainly of neonates ≥32 weeks, we intend to test its predictive performance in a new cohort of patients. Thus, neonates in the present study could be considered a “development cohort,” while those in the pending study would serve as a “validation cohort.” Depending upon accrual of patients in each gestational age subgroup, we may be able to create subgroup-specific algorithms to help guide the respiratory care of moderately preterm, late-preterm, and term neonates alike.

Our study was performed at tertiary care hospital with level 3 and 4 NICUs, but this technology may be useful at any hospital caring for newborns, including those with level 1 and 2 nursery capabilities. The technology is designed to be a point-of-care device that can be performed at the bedside by clinical staff without specialized training needed and give results within 15 min. Future studies will include clinical staff obtaining gastric aspirates after birth and using the point-of-care device to evaluate the sample.

## Data Availability

The raw data supporting the conclusions of this article will be made available by the authors, without undue reservation.
